# Fatigue in advanced cancer: a prospective controlled cross-sectional study

**DOI:** 10.1038/sj.bjc.6690236

**Published:** 1999-03

**Authors:** P Stone, J Hardy, K Broadley, A Kurowska, R A'Hern

**Affiliations:** Department of Palliative Medicine, The Royal Marsden NHS Trust, Sutton, Surrey, SM2 5PT, UK; Department of Computing and Statistics, The Royal Marsden NHS Trust, Sutton, Surrey, SM2 5PT, UK; Edenhall Marie Curie Centre, Edenhall Marie Curie Centre, Hampstead, London, NW3, UK

**Keywords:** fatigue, asthenia, neoplasms, palliative care, quality of life

## Abstract

Uncontrolled studies have reported that fatigue is a common symptom among patients with advanced cancer. It is also a frequent complaint among the general population. Simply asking cancer patients whether or not they feel fatigued does not distinguish between the ‘background’ level of this symptom in the community and any ‘excess’ arising as a result of illness. The aim of this study was to determine the prevalence of fatigue among palliative care inpatients in comparison with a control group of age and sex-matched volunteers without cancer. In addition, the correlates of fatigue were investigated. The prevalence of ‘severe subjective fatigue’ (defined as fatigue greater than that experienced by 95% of the control group) was found to be 75%. Patients were malnourished, had diminished muscle function and were suffering from a number of physical and mental symptoms. The severity of fatigue was unrelated to age, sex, diagnosis, presence or site of metastases, anaemia, dose of opioid or steroid, any of the haematological or biochemical indices (except urea), nutritional status, voluntary muscle function, or mood. A multivariate analysis found that fatigue severity was significantly associated with pain and dypnoea scores in the patients, and with the symptoms of anxiety and depression in the controls. The authors conclude that subjective fatigue is both prevalent and severe among patients with advanced cancer. The causes of this symptom remain obscure. Further work is required in order to determine if the associations reported between fatigue and pain and between fatigue and dyspnoea are causal or coincidental. © 1999 Cancer Research Campaign


British Journal of Cancer (1999) **79**(9/10), 1479–1486
© 1999 Cancer Research Campaign
Article no. bjoc.1998.0236

### Fatigue in advanced cancer: a prospective controlled cross-sectional study

**P Stone[1], J Hardy[1], K Broadley[1], AJ Tookman[2], A Kurowska[1] and R A'Hern[3]**

[1]Department of Palliative Medicine, [3]Department of Computing and Statistics, The Royal Marsden NHS Trust, Downs Road, Sutton, Surrey SM2 5PT, UK; [2]Edenhall Marie Curie Centre, Hampstead, London NW3, UK

**Summary** Uncontrolled studies have reported that fatigue is a common symptom among patients with advanced cancer. It is also a frequent complaint among the general population. Simply asking cancer patients whether or not they feel fatigued does not distinguish between the 'background' level of this symptom in the community and any 'excess' arising as a result of illness. The aim of this study was to determine the prevalence of fatigue among palliative care inpatients in comparison with a control group of age and sex-matched volunteers without cancer. In addition, the correlates of fatigue were investigated. The prevalence of 'severe subjective fatigue' (defined as fatigue greater than that experienced by 95% of the control group) was found to be 75%. Patients were malnourished, had diminished muscle function and were suffering from a number of physical and mental symptoms. The severity of fatigue was unrelated to age, sex, diagnosis, presence or site of metastases, anaemia, dose of opioid or steroid, any of the haematological or biochemical indices (except urea), nutritional status, voluntary muscle function, or mood. A multivariate analysis found that fatigue severity was significantly associated with pain and dypnoea scores in the patients, and with the symptoms of anxiety and depression in the controls. The authors conclude that subjective fatigue is both prevalent and severe among patients with advanced cancer. The causes of this symptom remain obscure. Further work is required in order to determine if the associations reported between fatigue and pain and between fatigue and dyspnoea are causal or coincidental.

**Keywords:** fatigue; asthenia; neoplasms; palliative care; quality of life

One of the key aspects to the good management of patients with advanced cancer is symptom control. In recent years it has been demonstrated that with attention to detail and good clinical practice, cancer pain can be controlled in a large proportion of patients (Twycross, 1994). Consequently, attention has now shifted towards the better management of other symptoms. Before improvements can occur it is first necessary to recognize which symptoms are most troublesome, and to devise simple methods by which they can be assessed. This is not a trivial problem, particularly when the patients concerned are severely debilitated. There have been no controlled studies using validated measures to assess the extent of fatigue in patients with advanced cancer who are no longer receiving active therapy. Uncontrolled studies in this population have reported fatigue prevalence to be anywhere between 33% and 89% (Walsh and Saunders, 1984; Coyle et al, 1990; Dunphy and Amesbury, 1990; McCarthy, 1990; Hardy et al, 1994; Donnelly and Walsh, 1995; Vainio and Auvinen, 1996). However, fatigue is also a common problem in the general population (Cox et al, 1987) and it is unclear how much of the fatigue experienced by the patients in these studies is in excess of that experienced by an otherwise healthy population of the same age.

The causes of cancer-related fatigue are also unknown. It is often assumed to be related to poor nutritional status (Neuenschwander and Bruera, 1996). Cachectic patients frequently have decreased muscle bulk and this might be expected to lead to physical weakness and easy fatiguability. Moreover, some authors have reported abnormal muscle electrophysiology in cancer patients even in the absence of cachexia (Bruera et al, 1988; Monga et al, 1997). However no study has yet demonstrated that these changes in muscle function are associated with increases in subjective fatigue. In the general population fatigue is frequently associated with psychological morbidity (Pawlikowska et al, 1994; Lawrie and Pelosi, 1995) and a similar association has been found in cancer patients receiving chemotherapy or radiotherapy (Bruera et al, 1989; Blesch et al, 1991; Morant, 1996; Smets et al, 1996). Other putative causes of cancer-related-fatigue include anaemia, biochemical abnormalities, the severity of other symptoms and non-specific 'stress'.

Fatigue is a difficult concept to define. We propose a simple distinction between the symptom of fatigue (a subjective sensation of feeling easily tired, weak or lacking in energy – which can only be measured by self-report instruments) and physical or muscular fatigue (a demonstrable decrement in strength over time). In this study we report the prevalence of 'severe subjective fatigue' (defined as a fatigue score greater than that experienced by 95% of an age-matched control group) in patients with advanced cancer. The clinical correlates of this symptom have also been investigated. In particular, the hypothesis that subjective fatigue in cancer patients is related to poor nutritional status and decreased muscle performance has been tested. This study represents the first prospective controlled study of fatigue in patients with advanced cancer who are no longer receiving active therapy (i.e. chemotherapy or radiotherapy).

*Received 19 May 1998*
*Revised 5 October 1998*
*Accepted 14 October 1998*

*Correspondence to:* P Stone, Trinity Hospice, 30 Clapham Common North Side, Clapham, London SW4 0RN, UK

### SUBJECTS AND METHODS

Inpatients with advanced cancer were recruited from three palliative care units between April 1996 and January 1997. Radiotherapy or chemotherapy within the previous 4 weeks, clinically apparent confusion, poor English language skills, disability or pain affecting the non-dominant hand or an estimated prognosis of less than 2 weeks were all exclusion criteria. The principal investigator visited each of the participating units weekly and approached all patients identified as being eligible to enter the study. In the absence of the principal investigator, due to holidays or illness, recruitment was suspended. Thus, although the patient group did not represent a consecutive series of eligible patients (i.e. it was a convenience sample), there is no reason to suspect a systematic bias in patient selection. Control subjects were age and sex-matched volunteers without cancer. They were recruited from the 'League of Friends' at the respective units and were subjected to the same inclusion and exclusion criteria as the patients. The protocol was approved by the relevant Scientific and Ethics Committees.

After informed consent had been obtained, the patients were asked to undergo a number of assessments (see below). Control subjects undertook all of the same assessments with the exception of the blood tests, which were omitted. The intention was to repeat all assessments (in both patients and controls) after an interval of at least 2 weeks. However, it should be noted that due to attrition not all patients were in fact reassessed.

### Questionnaires

The Fatigue Severity Scale (FSS) (Krupp et al, 1989) is a nine-item scale which has previously been used to assess fatigue in a number of medical conditions (Krupp et al, 1989; Krupp et al, 1990; Packer et al, 1991) but has not been previously validated or used in palliative care patients. Although other fatigue scales are available (Richardson, 1998) there is no universally accepted assessment instrument currently available. The FSS was chosen for this study because it is a short questionnaire (an important consideration in suck a sick patient population) which provides a simple unitary measure of global fatigue severity. Since the FSS had not previously been used in palliative care patients, the psychometric properties of the scale were reassessed as part of this study (see Results). The FSS is shown in Figure 1.

The European Organisation for Research and Treatment of Cancer Quality of Life Questionnaire (EORTC QLQ core 30) (Aaronson et al, 1993) is a well-validated 30-item quality of life questionnaire specifically designed for use in cancer patients. It has also been used in large demographic surveys of quality of life among the general population (Klee et al, 1997; Hjermstad et al, 1998). It consists of five functional scales (physical, role, cognitive, emotional and social functioning), three symptom scales (fatigue, pain and nausea/vomiting) and a number of single items assessing additional symptoms commonly reported by cancer patients (dyspnoea, loss of appetite, insomnia, constipation, diarrhoea and financial difficulties). Subjects are asked to rate each item on a four-point scale. Scores are then transformed onto a 0–100 scale: a higher score represents a higher (i.e. 'better') level of functioning, or a higher (i.e. 'worse') level of symptoms.

The Hospital Anxiety and Depression Scale (HADS) (Zigmond and Snaith, 1983) is a 14-item screening tool. It consists of separate scales for anxiety (HADA) and depression (HADD). The scale was developed for use amongst hospital inpatients and is designed to minimize the biological features of these conditions. It has been previously validated in patients with advanced cancer (Hopwood et al, 1991). Scores on each subscale can range between 0 (no symptoms of depression/anxiety) to 21 (numerous and severe symptoms). When the scale is used as a screening tool in medically sick populations a cut-off score of 11 or greater on either subscale is taken to indicate a 'probable case' of anxiety or depression (Snaith and Zigmond, 1994). One of the questions on the depression subscale (Item 8: 'I feel as if I am slowed down') may be construed as referring to fatigue and so to avoid confounding, this item was excluded from the analysis when correlations were sought with the FSS.

Three visual analogue scales (VAS) concerning tiredness, weakness and ability to concentrate were also used. While the other questionnaires inquired about symptoms in the previous week, the VAS specifically concerned symptoms experienced at the time of their completion. Each VAS was 100 mm in length and was anchored at either end by the descriptors 'not at all' and 'extremely'. Thus, higher scores represented a worse level of symptoms.

Choose a number from 1 to 7 that indicates your degree of agreement with the following statements where **1 indicates strongly disagree** and **7 indicates strongly agree**. Please answer the questions with reference to how you have been feeling **on average over the last week**.

| | Strongly disagree | | | | | | Strongly agree |
|---|---|---|---|---|---|---|---|
| 1. My motivation is lower when I am fatigued | 1 | 2 | 3 | 4 | 5 | 6 | 7 |
| 2. Exercise brings on my fatigue | 1 | 2 | 3 | 4 | 5 | 6 | 7 |
| 3. I am easily fatigued | 1 | 2 | 3 | 4 | 5 | 6 | 7 |
| 4. Fatigue interferes with my physical functioning | 1 | 2 | 3 | 4 | 5 | 6 | 7 |
| 5. Fatigue causes frequent problems for me | 1 | 2 | 3 | 4 | 5 | 6 | 7 |
| 6. My fatigue prevents sustained physical functioning | 1 | 2 | 3 | 4 | 5 | 6 | 7 |
| 7. Fatigue interferes with carrying out certain duties and responsibilities | 1 | 2 | 3 | 4 | 5 | 6 | 7 |
| 8. Fatigue is among my three most disabling symptoms | 1 | 2 | 3 | 4 | 5 | 6 | 7 |
| 9. Fatigue interferes with my work, family or social life | 1 | 2 | 3 | 4 | 5 | 6 | 7 |

**Figure 1** Fatigue severity scale

*© Cancer Research Campaign 1999*

### Tests of voluntary muscle function

Due to the nature of the study population it was not possible to subject volunteers to the standard tests of muscle strength and fatiguability. Many patients were confined to a bed or chair for most of the day and thus a portable bedside measure of voluntary muscle function had to be developed. Strength was determined with a hand-held grip dynamometer (Takei, Japan) using the best recording from three attempts with the non-dominant hand. Hand-grip dynamometry has previously been shown to be a reliable measure of voluntary muscle strength even in elderly subjects (Greig et al, 1994). Muscle fatiguability was determined by asking subjects to squeeze the investigator's fingers tightly for 10 s and then to record a measurement on the dynamometer. This process was repeated 18 times over a 6-min period. The speed of muscle recovery was assessed by asking subjects to register a maximal grip on the dynamometer at 1, 5 and 10 min after cessation of exercise. Strength was measured in Newtons ($1\ N = 1\ kg\ ms^{-2}$). The hand-grip dynamometer was periodically calibrated with free weights in order to check that it was reading true.

### Other assessments

The Body Mass Index (BMI) and the Mid Arm Muscle Circumference (MAMC) were calculated from the subjects' height, weight, mid-arm circumference and triceps skinfold thickness (measured with skinfold thickness calipers, Harpenden, UK). Patients (but not control subjects) also provided a full blood count, routine biochemistry and in male patients without prostate cancer; testosterone, leutinizing hormone (LH) and sex hormone binding globulin (SHBG). Blood specimens for these latter tests were taken before 11 am in order to minimize the effects of diurnal variation.

### Statistical methods

Median survival was calculated using the Kaplan-Meier method. Measurement scales could typically only take a defined number of discrete values; non-parametric methods of analysis were therefore employed, e.g. correlation was assessed using the Spearman Rank Correlation Coefficient (Rs) and the Mann-Whitney *U*-test was used for comparing groups. The calculation of Spearman's Rank correlation included a correction for ties (a tie occurring when two or more data items had the same value) (Zar, 1984*a*). Multiple regression analysis (Zar, 1984*b*) was used to identify subsets of factors that were independently predictive of fatigue. Analysis was undertaken using the MINITAB program (Minitab Inc, State College, PA, USA).

### RESULTS

### Description of the study population

Of 122 patients initially approached, 19 refused consent and 8 were subsequently excluded because of protocol violations. None of the controls refused consent. Data from 95 patients and 98 control subjects were included in the final analysis. There were no significant differences in either age or sex between the two groups. Despite being a convenience sample the control group appeared to be reasonably representative of the UK adult population, albeit with a moderate excess of females (57% F; 43% M). Over 90% of the control group were white, with 49% (48/98) being overweight or obese and 50% (49/98) having at least one concomitant medical problem (e.g. arthritis, chronic airflow limitation or hypertension). The median survival of patients after entry into the study was 50 days (2–776), 71% (67/95) of patients were on strong opioids and 67% (64/95) were taking corticosteroids. A wide variety of tumour types were represented, with the majority suffering from lung ($n$ = 26), breast ($n$ = 24) or prostate cancer ($n$ = 13). The majority of patients had metastatic disease with bone (48/95), liver (18/95) and lung (9/95) being the commonest sites of spread. The median Eastern Cooperative Oncology Group (ECOG) performance status of the patients was 3 (i.e. confined to bed or chair for greater than 50% of the day).

### Reliability and validity of the assessment instruments

The internal consistency of the FSS was found to be acceptable in both patients (Cronbach's alpha = 0.94) and controls (Cronbach's alpha = 0.88). Test-retest reliability of the scale was assessed by calculating the measurement error (or within subject standard deviation), a standard method for determining instrument reliability (Bland and Altman, 1996). Among the 87 control subjects who completed the FSS on two occasions a median of 21 days apart, the measurement error was 4.7. The underlying structure of the FSS was further investigated by using principal components analysis. A single factor solution explained 76% of the variation in fatigue scores. The item loadings of the individual questions were all in excess of 0.67. These results suggest that the sum of the responses to the nine items on the FSS provides an adequate summary of a single underlying factor. Although no 'gold standard' assessment instrument exists with which to compare the performance of the FSS, its validity as a measure of fatigue was supported by the significant correlation that was found between the FSS and the EORTC Fatigue subscale (Rs = 0.73 in patients and 0.61 in controls, $P < 0.001$ in both cases).

The reliability of the voluntary muscle function tests was checked in the 87 control subjects who completed two assessments. The measurement error for the grip strength readings was 17 N. The tests of muscle fatigue showed a systematic difference between the two assessments, with control subjects becoming less fatigued at the second visit. This probably represented a 'learning effect' due to the controls being more familiar with what was required of them when they attended for a repeat assessment. It should be noted that no such learning effect was apparent in the advanced cancer patients, perhaps because any improvements due to familiarity were counterbalanced by a decline in physical performance.

### Differences between groups at the first assessment

Both patients and controls complained of at least some degree of fatigue (Table 1), but the severity of the symptom in the patients was much worse. The prevalence of 'severe subjective fatigue' (defined as a score on the FSS of greater than the 95th percentile of controls) among palliative care inpatients was 75% (Figure 2).

Patients' grip strength was significantly less than that of control subjects: 201 N vs 279.5 N ($P < 0.001$). Patients' muscles were also more easily fatigued (Figure 3). After 6 min exercise the patients' strength had declined to 61% of the pre-exercise level, whereas the control subjects' strength had fallen to 68% of the initial value. Although not large, this difference was highly significant ($P < 0.001$). There was no difference in the speed of recovery of muscle strength at the end of exercise between the two groups.

© Cancer Research Campaign 1999

**Table 1** Differences between patients and controls: median values (range)

| | Patients | Controls | *P* value |
|---|---|---|---|
| Number | 95 | 98 | |
| Age in years | 67 (30–89) | 68 (41–85) | NS |
| Gender % female | 57 | 62 | NS |
| Measures of subjective fatigue (higher values indicate greater fatigue) | | | |
| FSS | 54 (9–63) | 20 (9–51) | < 0.001 |
| EORTC fatigue scale | 67 (0–100) | 11 (0–56) | < 0.001 |
| VAS tired (mm) | 62 (0–100) | 11 (0–74) | < 0.001 |
| VAS weak (mm) | 61.5 (0–100) | 4 (0–52) | < 0.001 |
| VAS concentration (mm) | 56 (0–97) | 3 (0–50) | < 0.001 |
| Measures of muscle function (6-min grip fatigue and 10-min grip recovery are expressed as a proportion of the initial strength) | | | |
| Grip strength (N) | 201 (0–495) | 279.5 (147–519.5) | < 0.001 |
| 6 min grip fatigue | 0.61 (0–1.00) | 0.68 (0.43–1.01) | < 0.001 |
| 10 min grip recovery | 0.94 (0.63–1.23) | 0.95 (0.74–1.26) | NS |
| Measures of nutritional status (higher values indicate better nutrition) | | | |
| BMI (kg/m$^2$) | 22.4 (14.0–35.6) | 25.1 (18.1–40.4) | < 0.001 |
| MAMC (cm) | 21.5 (14.7–31.0) | 23.4 (18.2–31.5) | < 0.001 |
| Measures of psychological distress (higher scores indicate more symptoms) | | | |
| HAD-A | 8 (0–21) | 4 (0–17) | < 0.001 |
| HAD-D | 9 (0–18) | 1 (0–13) | < 0.001 |
| Functional subscales and global quality of life of EORTC QLQc30 (higher scores indicate better function) | | | |
| Physical function | 40 (0–100) | 100 (40–100) | < 0.001 |
| Role function | 17 (0–100) | 100 (17–100) | < 0.001 |
| Emotional function | 67 (0–100) | 92 (42–100) | < 0.001 |
| Cognitive function | 50 (0–100) | 83 (50–100) | < 0.001 |
| Social function | 30 (0–100) | 100 (17–100) | < 0.001 |
| Global quality of life | 33 (0–100) | 83 (33–100) | < 0.001 |
| Symptom subscales of EORTC QLQc30 (higher scores indicate more severe symptoms) | | | |
| Nausea and vomiting | 17 (0–100) | 0 (0–33) | < 0.001 |
| Pain | 67 (0–100) | 0 (0–83) | < 0.001 |
| Dyspnoea | 67 (0–100) | 0 (0–67) | < 0.001 |
| Insomnia | 33 (0–100) | 0 (0–100) | < 0.001 |
| Anorexia | 67 (0–100) | 0 (0–67) | < 0.001 |
| Constipation | 33 (0–100) | 0 (0–67) | < 0.001 |
| Diarrhoea | 0 (0–100) | 0 (0–67) | < 0.001 |
| Financial affairs | 0 (0–100) | 0 (0–33) | < 0.001 |

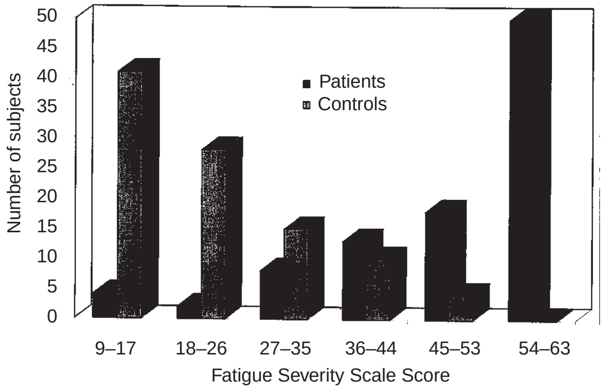

**Figure 2** Distribution of fatigue severity scale scores

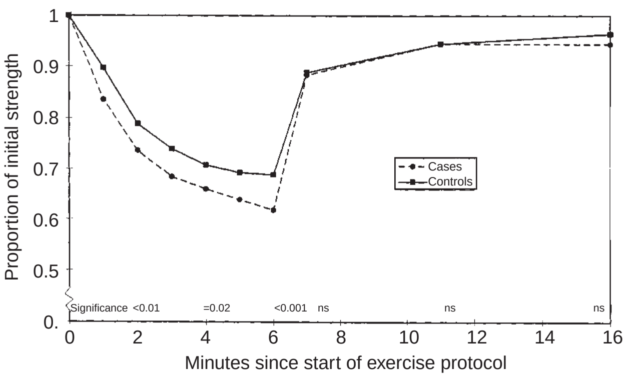

**Figure 3** Grip fatigue expressed as a proportion of initial grip strength

Many of the patients were undernourished. If a MAMC of less than the 15th percentile of published normal values (Frisancho, 1974) is taken as indicating nutritional depletion, then 39/95 (41%) of patients and only 10/98 (10%) of controls were malnourished ($P < 0.001$).

As expected, patients were more symptomatic than controls. Using the recommended cut-offs for case-detection on the HADS, 27/95 (29%) patients and 6/98 (6%) controls were 'probable cases' of anxiety and 33/95 (35%) patients and 1/98 (1%) controls were 'probable cases' of depression. There were also significant differences in all of the domains assessed by the EORTC QLQ core 30.

Routine haematological and biochemical blood tests were taken from 88/95 (93%) of the patients. Hormonal assays were undertaken in 21/28 (75%) of male patients without prostate cancer. The proportion of patients with blood results outside of the normal range is shown in Table 2. There was a high prevalence of

 *© Cancer Research Campaign 1999*

**Table 2** Frequency of abnormal blood results in patients

| | Normal range | Low *n* (%) | Normal *n* (%) | High *n* (%) |
|---|---|---|---|---|
| Testosterone nmol $l^{-1}$ | 10–30 | 11 (52) | 10 (48) | 0 (0) |
| LH IU $l^{-1}$ | 2–12 | 2 (10) | 16 (76) | 3 (14) |
| Sodium mmol $l^{-1}$ | 135–145 | 27 (31) | 61 (69) | 0 (0) |
| Potassium mmol $l^{-1}$ | 3.5–5.0 | 8 (9) | 77 (88) | 3 (3) |
| Urea mmol $l^{-1}$ | 2.5–6.4 | 6 (7) | 41 (47) | 41 (46) |
| Glucose mmol $l^{-1}$ | 3.9–5.9 | 4 (5) | 33 (42) | 41 (53) |
| Calcium mmol $l^{-1}$ | 2.2–2.60 | 3 (3) | 70 (80) | 15 (17) |
| Magnesium mmol $l^{-1}$ | 0.65–1.05 | 5 (8) | 51 (89) | 2 (3) |
| Phosphate mmol $l^{-1}$ | 0.81–1.45 | 7 (13) | 39 (74) | 7 (13) |
| Albumin g $l^{-1}$ | 30–50 | 30 (34) | 58 (66) | 0 (0) |
| Haemoglobin g $dl^{-1}$ | M 13.5–17.5<br>F 11.5–15.5 | 54 (62) | 33 (38) | 0 (0) |

Note: Not all patients had all blood tests. Blood specimens were not taken from healthy control subjects.

anaemia, hypogonadism and hyponatraemia (all of which may cause fatigue). However, no association was demonstrated between the extent of these abnormalities and the level of fatigue experienced by the patients. The only significant association was between fatigue severity and blood urea (Rs = 0.27, $P < 0.05$).

### Changes occurring between the first and second assessments (in the patients)

Of the 95 patients who completed a baseline assessment only 43 were able to complete a second assessment 2 weeks later (22 patients had died, 14 were lost to follow-up, nine were too ill to continue, four withdrew their consent, two had since developed weakness in the non-dominant arm and one was too confused to complete the questionnaires). Generally, the results of the second assessment were in keeping with the results of the baseline measurements. The only significant changes to have occurred were a reduction in grip strength (median change of 15 N, $P < 0.01$), a reduction in the MAMC (median change 0.8 cm, $P < 0.01$) and an improvement in insomnia (median change 16.5, $P < 0.05$).

### Correlates of subjective fatigue at the first assessment

### *Patients*

There were no significant associations between fatigue severity and age, sex, diagnosis, the presence or site of metastases or the dose of opioid or steroid (Table 3). Although patients were malnourished and had evidence of impaired voluntary muscle function, there was no association between the degree of these abnormalities and the level of their fatigue. Similarly, there were no significant associations between the FSS and either subscale of the HADS.

Fatigue severity was, however, significantly associated with a number of the domains of the EORTC QLQ core 30 (physical, role and social functioning, pain, dyspnoea, insomnia, anorexia, constipation and global quality of life).

### *Controls*

As in the patient group, there was no association between the FSS and the age, sex, nutritional status or muscle function of the volunteers. In most respects the findings in the control group were very similar to the findings in the patient group. The most obvious differences between the two groups was the finding of a highly significant association between the FSS and the HADS in the control group but the lack of such a relationship in the patients. This difference in correlation coefficients between the two groups was significant ($P < 0.05$).

### *Multiple regression analyses*

The correlates of subjective fatigue were also explored by using multiple regression techniques. The FSS was taken as the dependent variable. The other study data were entered into the analyses as independent variables. Certain data was, however, excluded:

- The other measures of subjective fatigue (i.e. the EORTC Fatigue subscale and the three VAS pertaining to fatigue) – these were clearly not independent variables.
- The ECOG performance status, the EORTC physical, role and social functioning subscales and the EORTC global quality of life subscale – these variables were felt to be more likely to represent the effects of rather than the causes of fatigue.

In the patient group (Table 4), 30% of the variance in fatigue scores could be explained by the combination of the EORTC pain and dyspnoea scores. In the controls, 17% of the variance in fatigue scores could be explained by the total HADS score (minus question 8).

### DISCUSSION

In this study we were able to demonstrate that subjective and objective fatigue can be assessed in even very sick patients with advanced cancer. Most patients were able to complete the nine-item FSS with little difficulty. The necessity of using a control group was demonstrated by the fact that many of the healthy volunteers reported at least some degree of fatigue over the preceding week. However, by defining 'severe subjective fatigue' as a score greater than that experienced by 95% of an age-matched control group, we were able to account for this 'background' level of fatigue.

As well as feeling more fatigued, patients had reduced voluntary muscle strength and a diminished ability to maintain strength with repeated contractions. It had been uncertain whether the test of voluntary muscle fatigue devised for this research would be sensitive enough to detect these differences. Our results indicate that this exercise protocol was both practical to use and was able to discriminate between healthy volunteers and cancer patients.

© Cancer Research Campaign 1999 

**Table 3** Correlation (Spearman Rank) between the FSS and other study variables

| | Patients | Controls |
|---|---|---|
| Age | 0.00 | –0.08 |
| Correlation with measures of muscle function | | |
| Corrected grip strength | –0.16 | –0.08 |
| 6-min grip fatigue | –0.02 | 0.04 |
| 10-min grip recovery | 0.09 | 0.00 |
| Correlation with measures of psychological distress | | |
| HAD-A | 0.16 | 0.40*** |
| HADD (-Q8) | 0.16 | 0.38*** |
| HADT (-Q8) | 0.18 | 0.44*** |
| Correlation with EORTC QLQc30 subscales: functional abilities and quality of life | | |
| Physical functioning | –0.43*** | –0.12 |
| Role functioning | –0.42*** | –0.56*** |
| Emotional functioning | –0.09 | –0.04 |
| Cognitive functioning | –0.15 | 0.04 |
| Social functioning | –0.44*** | –0.47*** |
| Global quality of life | –0.35*** | –0.24* |
| Correlation with symptom subscales of EORTC QLQc30 | | |
| Fatigue | 0.71*** | 0.61*** |
| Nausea and vomiting | 0.08 | 0.13 |
| Pain | 0.45*** | 0.30** |
| Dyspnoea | 0.50*** | 0.23* |
| Insomnia | 0.41*** | 0.27** |
| Anorexia | 0.46*** | 0.30** |
| Constipation | 0.31** | 0.14 |
| Diarrhoea | 0.10 | 0.11 |
| Financial difficulties | 0.24 | –0.01 |
| Correlation with measures of nutritional status | | |
| BMI | 0.02 | 0.01 |
| MAMC | –0.05 | –0.04 |

*$P < 0.05$, **$P < 0.01$, ***$P < 0.001$,

**Table 4** Multiple regression analysis in patients and controls

| | | Patients | | Controls |
|---|---|---|---|---|
| Step | | 1 | 2 | 1 |
| Intercept | | 42.1 | 33.7 | 16.7 |
| EORTC-Dyspnoea | T-value | 4.0 | 4.5 | – |
| | Slope | 0.14 | 0.15 | – |
| EORTC-Pain | T-value | | 4.4 | – |
| | Slope | | 0.15 | – |
| HADT-8 | T-value | – | – | 4.4 |
| | Slope | – | – | 0.9 |
| $R^2$ | | 15 | 30 | 17 |

Independent variables included in analysis: all EORTC QLQc30 subscales except physical, role and social functioning, fatigue and global quality of life; HADA, HADD(-Q8) and HADT (-Q8); BMI, MAMC; tests of voluntary muscle function; blood results

However, despite the differences in muscle strength and fatiguability that were demonstrated between these two groups, there was no relationship between these tests and the measures of subjective fatigue. This suggests that the symptom of fatigue, although often chiefly experienced as a subjective sensation of physical tiredness, is not primarily caused by muscular weakness/fatigue. Other authors have reported a similar poor relationship between subjective and objective measures of fatigue in patients with chronic fatigue syndrome (Wearden and Appleby, 1996; Lawrie et al, 1997). However, very few studies have specifically investigated this relationship in cancer patients (Dimeo et al, 1997; Monga et al., 1997).

Cachexia and fatigue are often thought to be closely related and we had expected to find that the more cachectic patients would also experience more fatigue. Bruera and coworkers (Bruera et al, 1989) failed to find any such association in patients with advanced breast cancer, but concluded that this was probably due to their studying a relatively well nourished group of patients. In our study, despite a high prevalence of cachexia we were still unable to find an association between fatigue and nutritional status. This suggests that a clearer distinction should be made between these two symptoms and that they probably have different aetiologies.

In the general population symptoms of fatigue are often associated with psychological morbidity (Pawlikowska et al, 1994). We found a significant association between these two symptoms in our control group but rather surprisingly, failed to find any such association in the cancer patients. This is in contrast to a number of

 *© Cancer Research Campaign 1999*

other studies which have found a positive relationship between these two constructs in patients with cancer (Bruera et al, 1989; Morant et al, 1993; Smets et al, 1996). The discrepancy may partly be explained by the nature of our patient sample. All of the patients in our study had advanced disease and most had multiple physical problems and a very short prognosis. Under these circumstances it is possible (indeed probable) that fatigue has a different origin to that occurring in patients with earlier stage disease or indeed in the general population. The nature of the association between fatigue and psychological morbidity is clearly a complex one. Fatigue may be a cause or an effect of psychological distress or the two symptoms may both be related to other confounding factors (Visser and Smets, 1998).

The factors which appeared to be most associated with fatigue in the patients were the severity of their other physical symptoms (i.e. pain, dyspnoea, insomnia, anorexia and constipation). It could be postulated that fatigue is a result of non-specific ‘stress’ (Aistars, 1987), and that in patients with advanced cancer physical symptoms are a major source of such stress. If this were the case, then measures designed to relieve these other symptoms might be expected to incidentally improve fatigue. To test this hypothesis we are currently undertaking a study to investigate the effect of improved pain control upon fatigue levels.

Of all symptoms, dyspnoea appeared to be the most consistently associated with fatigue. To a certain extent this is unfortunate, because dyspnoea is already recognized as being a difficult symptom to treat (Higginson and McCarthy, 1989). It is unclear whether the relationship between these two symptoms is causal or coincidental. Dyspnoea may lead to fatigue by limiting exercise tolerance. Some patients, particularly those with lung cancer, may have difficulty in distinguishing between these two symptoms (Brown et al, 1986). It is also possible that in some patients both fatigue and dyspnoea are caused by the same underlying process. This is a potentially fruitful area for future research. We recommend that future studies into the management of dyspnoea should also include a validated fatigue assessment (and vice versa) so that the relationship between these two symptoms might be further elucidated.

The results of the multiple regression analyses indicated that a large proportion of the variance in fatigue scores remained unaccounted for. This could partly be a reflection of our subject population. Although the patients had a variety of different tumour types and varied in both age and sex, they were nearly all suffering from severe fatigue and a large number of other physical and mental symptoms. The magnitude of a correlation coefficient is partly dependent upon the range over which the study variables are measured (the wider the range, the greater the potential correlation). Thus, the fact that most of our patients were clustered at one extreme or the other on most of the assessment scales which were used, will have resulted in the correlation coefficients being lower than if a broader population of cancer patients had been studied. However, the use of a more heterogenous population would have cast doubts over the applicability of the results to any particular patient group. As has already been indicated, it may well be that the origins of fatigue in the early stages of cancer are significantly different from the causes of fatigue in advanced disease.

Fatigue is a difficult symptom to study in any group of patients but particularly in those with advanced cancer. The research methodology described in this paper was found to be both practical and reliable in even very sick patients and could be adopted for use in future palliative care studies. It is hoped that recognizing the extent and severity of this symptom in advanced cancer will be the first step towards improving its management in the future.

### ACKNOWLEDGMENTS

The authors would like to thank Professor M Richards for his help in the preparation of this manuscript.

### REFERENCES

Aaronson NK, Ahmedzai S, Bergman B, Bullinger M, Cull A, Duez NJ, Filiberti A, Flechtner H, Fleishman SB, de Haes JC, Kaasa S, Klee M, Osoba D, Razavi D, Rofe PB, Schraub S, Sneeuw K, Sullivan M and Takeda F (1993) The European Organization for Research and Treatment of Cancer QLQ-C30: a quality-of-life instrument for use in international clinical trials in oncology. *J Natl Cancer Inst* **85**: 365–376

Aistars J (1987) Fatigue in the cancer patient: a conceptual approach to a clinical problem. *Oncol Nurs Forum* **14**: 25–30

Bland J and Altman D (1996) Measurement error. *BMJ* **312**: 1654

Blesch KS, Paice JA, Wickham R, Harte N, Schnoor DK, Purl S, Rehwalt M, Kopp PL, Manson S, Coveny SB, McHale M and Cahill M (1991) Correlates of fatigue in people with breast or lung cancer. *Oncology Nursing Forum* **18**: 81–87

Brown M, Carrieri V, Janson-Bjerklie S and Dodd M (1986) Lung cancer and dyspnoea: the patient’s perception. *Oncology Nursing Forum* **13**: 19–24

Bruera E, Brenneis C, Michaud M, Jackson PI and MacDonald RN (1988) Muscle electrophysiology in patients with advanced breast cancer. *J Natl Cancer Inst* **80**: 282–285

Bruera E, Brenneis C, Michaud M, Rafter J, Magnan A, Tennant A, Hanson J and Macdonald RN (1989) Association between asthenia and nutritional status, lean body mass, anemia, psychological status, and tumor mass in patients with advanced breast cancer. *J Pain Symptom Manage* **4**: 59–63

Cox B, Blaxter M, Buckle A, Fenner N, Golding J, Huppert F, Nickson J, Roth M, Stark J, Wadsworth M and Whichelow M (1987) *The Health and Lifestyle Survey*. Health Promotion Research Trust: London

Coyle N, Adelhardt J, Foley KM and Portenoy RK (1990) Character of terminal illness in the advanced cancer patient: pain and other symptoms during the last four weeks of life. *J Pain Symptom Manage* **5**: 83–93

Dimeo F, Stieglitz RD, NovelliFischer U, Fetscher S, Mertelsmann R and Keul J (1997) Correlation between physical performance and fatigue in cancer patients. *Annals Of Oncology* **8**: 1251–1255.

Donnelly S and Walsh D (1995) The symptoms of advanced cancer. *Semin Oncol* **22**: 67–72

Dunphy K and Amesbury B (1990) A comparison of hospice and homecare patients: patterns of referral, patient characteristics and predictors of place of death. *Palliat Med* **4**: 105–111

Frisancho AR (1974) Triceps skinfold and upper arm muscle size norms for assessment of nutritional status. *Am J Clin Nutr* **27**: 1052

Greig CA, Young A, Skelton DA, Pippet E, Butler FM and Mahmud SM (1994) Exercise studies with elderly volunteers. *Age Ageing* **23**: 185–189.

Hardy JR, Turner R, Saunders M and R AH (1994) Prediction of survival in a hospital-based continuing care unit. *Eur J Cancer* **30a**: 284–288

Higginson I and McCarthy M (1989) Measuring symptoms in terminal cancer: are pain and dyspnoea controlled? *Journal of the Royal Society of Medicine* **82**: 264–267.

Hjermstad M, Fayers P, Bjordal K and Kaasa S (1998) Health-related quality of life in the general Norwegian population assessed by the European Organisation for Research and Treatment of Cancer Core Quality of Life Questionnaire: The QLQ = C30 (+3). *J Clin Oncol* **16**: 1188–1196

Hopwood P, Howell A and Maguire P (1991) Screening for psychiatric morbidity in patients with advanced breast cancer: validation of two self-report questionnaires. *British Journal of Cancer* **64**: 353–356

Klee M, Groenvold M and Machin D (1997) Quality of Life of Danish Women: Population-based norms for the EORTC-QLQ-C30. *Qual Life Res* **6**: 27–34

Krupp LB, LaRocca NG, Muir J and Steinberg AD (1990) A study of fatigue in systemic lupus erythematosus. *J Rheumatol* **17**: 1450–1452

Krupp LB, LaRocca NG, Muir Nash J and Steinberg AD (1989) The fatigue severity scale. Application to patients with multiple sclerosis and systemic lupus erythematosus. *Arch Neurol* **46**: 1121–1123

Lawrie S, MacHale S, Power M and Goodwin G (1997) Is the chronic fatigue syndrome best understood as a primary disturbance of the sense of effort? *Psychological Medicine* **27**: 995–999

© Cancer Research Campaign 1999

Lawrie S and Pelosi A (1995) Chronic fatigue syndrome in the community: prevalence and associations. *British Journal of Psychiatry* **166**: 793–797

McCarthy M (1990) Hospice patients: a pilot study in 12 services. *Palliat Med* **4**: 93–104

Monga U, Jaweed M, Kerrigan AJ, Lawhon L, Johnson J, Vallbona C and Monga TN (1997) Neuromuscular fatigue in prostate cancer patients undergoing radiation therapy. *Arch Phys Med Rehabil* **78**: 961–966

Morant R (1996) Asthenia: an important symptom in cancer patients. *Cancer Treat Rev* **22** Suppl A: 117–122

Morant R, Stiefel F, Berchtold W, Radziwill A and Riesen W (1993) Preliminary results of a study assessing asthenia and related psychological and biological phenomena in patients with advanced cancer. *Support Care Cancer* **1**: 101–107

Neuenschwander H and Bruera E (1996) Asthenia–Cachexia. In *Cachexia–Anorexia in Cancer Patients*. Bruera E and Higginson I. (eds) pp 57–75. Oxford University Press Oxford

Packer TL, Martins I, Krefting L and Brouwer B (1991) Activity and post-polio fatigue. *Orthopedics* **14**: 1223–1226

Pawlikowska T, Chalder T, Hirsch S, Wallace P, Wright D and Wessely S (1994) A population based study of fatigue and psychological distress. *BMJ* **308**: 743–746

Richardson A (1998) Measuring fatigue in patients with cancer. *Support Care Cancer* **6**: 94–100

Smets EM, Garssen B, Cull A and de Haes JC (1996) Application of the multidimensional fatigue inventory (MFI-20) in cancer patients receiving radiotherapy. *Br J Cancer* **73**: 241–245

Snaith R and Zigmond A (1994) *The Hospital Anxiety and Depression Scale – Manual*. NFER Nelson: Windsor

Twycross R (1994) *Pain Relief in Advanced Cancer*. Churchill Livingstone: London

Vainio A and Auvinen A (1996) Prevalence of symptoms among patients with advanced cancer: an international collaborative study. Symptom Prevalence Group. *J Pain Symptom Manage* **12**: 3–10

Visser MRM and Smets EMA (1998) Fatigue, depression and quality of life in cancer patients: how are they related? *Supportive Care in Cancer* **6**: 101–108

Walsh TD and Saunders CM (1984) Hospice care: the treatment of pain in advanced cancer. *Recent Results Cancer Res* **89**: 201–211

Wearden AJ and Appleby L (1996) Research on cognitive complaints and cognitive functioning in patients with chronic fatigue syndrome (CFS): What conclusions can we draw? *J Psychosom Res* **41**: 197–211

Zar J (1984*a*) Simple Linear Correlation. *Biostatistical Analysis*, pp 318–320. Prentice Hall: New Jersey

Zar J (1984*b*) Multiple Regression and Correlation. In *Biostatistical Analysis*, pp 328–360. Prentice Hall: New Jersey

Zigmond AS and Snaith RP (1983) The hospital anxiety and depression scale. *Acta Psychiatr Scand* **67**: 361–370

*© Cancer Research Campaign 1999*
